# The role of TLR-4 in chemoresistance of cancer

**DOI:** 10.1007/s12672-025-02509-z

**Published:** 2025-05-22

**Authors:** Yuhua Li, Tianle Tang, Yang Sun, Gui’e Chen, Xinrong Yuan, De Cai

**Affiliations:** 1 Department of Pharmacy, Affiliated Hospital of Guangdong Medical University, Zhanjiang, 524001 Guangdong People’s Republic of China; 2 Department of Pharmacy, The First Naval Force Hospital of Southern Theatre Command, Zhanjiang, 524005 Guangdong People’s Republic of China; 3 Xijing Hospital, The Fourth Military Medical University, Xi’an, 710032 Shaanxi People’s Republic of China; 4 Key Laboratory of Gastrointestinal Pharmacology of Chinese Materia Medica of the State Administration of Traditional Chinese Medicine, Department of Pharmacology, School of Pharmacy, The Fourth Military Medical University, Xi’an, 710032 Shaanxi People’s Republic of China; 5 Department of Gynecology and Obstetrics, The First Naval Force Hospital of Southern Theatre Command, Zhanjiang, 524005 Guangdong People’s Republic of China

**Keywords:** Toll like receptor 4 (TLR-4), Cancer, Chemoresistance

## Abstract

Chemotherapy, which aims to eradicate tumor cells and enhance patient survival, is a prevalent approach for tumor treatment. Nevertheless, recurrence and drug resistance resulting from consecutive chemotherapy regimens have emerged as significant factors contributing to the high fatality rates among cancer patients. Numerous studies have revealed that chemicals discharged by injured and deceased cells can trigger the host repair program mediated by toll-like receptor-4 (TLR-4), enhancing tumor resistance. TLR-4 is not only expressed in immune cells but also in various malignant tumor cells, especially inflammation-associated tumor cells, and plays a crucial role in tumor formation, development, and chemoresistance. Endogenous ligands are released upon the killing of tumor cells by chemotherapy drugs, binding to and activating TLR-4, subsequently activating downstream NF-κB and other essential molecules, leading to the release of multiple factors associated with tumor proliferation and invasion, creating a microenvironment conducive to local recurrence and metastasis, and promoting tumor progression and drug resistance. This review assessed studies on the resistance of several tumor cells to commonly utilized anticancer treatments induced by TLR-4 to better comprehend the phenomena and mechanism of TLR-4-dependent resistance, as well as to put forward suggestions and insights for overcoming tumor resistance.

## Introduction

Cancer poses a significant and severe threat to global public health. According to the World Health Organization's (WHO) 2024 projections, cancer is ranked as either the first or second leading cause of death among individuals under the age of 70 in 185 countries. In 2022, it was estimated that approximately 20 million new cancer cases emerged globally [1].

Chemotherapy is a crucial cancer treatment method. Its primary objective is to eliminate tumor cells and enhance patient survival. Nevertheless, chemotherapy agents lack the ability to selectively target tumor cells while sparing healthy cells. This non-discriminatory action causes collateral damage to normal tissues, resulting in various side effects that may undermine the overall treatment effectiveness. To date, chemotherapy remains one of the fundamental therapies for treating tumors. In cases of advanced, refractory, or metastatic cancers, as well as malignancies lacking specific molecular targets, chemotherapeutic agents often serve as the main or only treatment option. Paradoxically, chemotherapy has dual effects: it can suppress tumor growth locally but may also inadvertently promote tumor metastasis and chemoresistance [2, 3].

Given the importance of chemotherapy in cancer treatment and the challenges it faces, this paper focuses on the *TLR-4* activation mechanisms underlying chemotherapy-induced resistance in specific cancer types. TLR-4 is a vital pattern recognition receptor that identifies highly conserved pathogen-associated molecular patterns (PAMPs) and damage-associated molecular patterns (DAMPs) [4–6]. It has a substantial function in innate immunity, acting as a bridge between innate and adaptive immunity [7, 8]. Moreover, TLR-4 is expressed not only in immune cells but also highly in various malignant tumor cells, especially those associated with inflammation. Its presence in these cells significantly impacts tumor occurrence, development, and drug resistance [9–12].

*TLR-4* has a pivotal position in immune regulation, and its targeted therapy holds broad application prospects. In inflammatory diseases like sepsis and atherosclerosis, inhibiting TLR-4 signal transduction effectively alleviates excessive inflammatory responses. Similarly, in viral infectious diseases such as influenza and COVID-19, blocking *TLR-4* activity significantly mitigates disease progression. In cancer treatment, activating *TLR-4* can overcome tumor immune tolerance and enhance anti-tumor immune responses. For example, specific *TLR-4* agonists induce the polarization of tumor-associated macrophages (TAMs) toward the M1 phenotype, thereby enhancing the body's immune-mediated cytotoxicity against tumor cells. Based on structural design, disaccharide lipid A mimetics (DLAMs) precisely modulate *TLR-4* activity. Among these compounds, antagonists (βα-DLAMs) and agonists (αα-DLAMs and ββ-DLAMs) exhibit potent biological activity and high specificity, enabling precise regulation of the TLR-4 signaling pathway [13].

When *TLR-4* binds exogenous ligands like bacterial lipopolysaccharide or viral fusion proteins, it recruits *Myeloid Differentiation Primary Response 88* (*MyD88*), the *Toll/Interleukin-1 Receptor* (*TIR*), and *TIR-Domain-Containing Adaptor Inducing Interferon-β* (*TRIF*), triggering *MyD88*-dependent and *MyD88*-independent pathways [14, 15]. These two pathways exert distinct effects on chemoresistance.

The *MyD88*-dependent pathway functions as follows: *MyD88* activation recruits *Interleukin-1 Receptor-Associated Kinase 1* (*IRAK1*) and *Interleukin-1 Receptor-Associated Kinase 4* (*IRAK4*). *IRAK4* phosphorylates *IRAK1*, causing it to dissociate from the *MyD88-IRAK* complex. Activated *IRAK1* then recruits and phosphorylates *TNF Receptor-Associated Factor 6* (*TRAF6*). *TRAF6* undergoes polyubiquitination and activates *TGF-β-Activated Kinase 1* (*TAK1*). *TAK1* phosphorylates the IκB kinase (IKK) complex, leading to IκB-α phosphorylation and degradation. This releases *Nuclear Factor-κB* (*NF-κB*), allowing it to translocate to the nucleus. In the nucleus, *NF-κB* binds to the promoter regions of genes encoding pro-inflammatory cytokines such as *Tumor Necrosis Factor-α* (*TNF-α)* and *Interleukin-6* (*IL-6*), initiating their transcription and production. Regarding chemoresistance, the production of *TNF-α* and *IL-6* contributes to a tumor-promoting microenvironment. Chronic exposure to *TNF-α* in the tumor microenvironment can activate survival pathways in tumor cells, resulting in chemoresistance. Similarly, *IL-6* binding to its receptor on tumor cells activates the *Janus Kinase-Signal Transducer and Activator of Transcription 3* (*JAK-STAT3*) pathway, promoting cell proliferation, survival, and angiogenesis, all of which contribute to chemoresistance. For instance, *STAT3* activation upregulates anti-apoptotic proteins (e.g., *Bcl-2)* and facilitates cell cycle progression through the regulation of cyclins and cyclin-dependent kinases [6, 16–18].

In contrast, the *MyD88*-independent pathway works differently: *TLR-4* recruits *TIR-Domain-Containing Adaptor Inducing Interferon-β* (*TRIF*) through its Toll/IL-1 receptor (TIR) domain. *TRIF* recruits *TNF Receptor-Associated Factor 3* (*TRAF3*), which activates *TANK-Binding Kinase 1* (*TBK1*) and *IKKi*. These kinases phosphorylate *Interferon Regulatory Factor 3* (*IRF3*), leading to its dimerization. Phosphorylated *IRF3* translocates to the nucleus, binds to the promoter regions of type I interferon-encoding genes (e.g., *IFN-β*), and initiates their transcription, producing type I interferon [13, 14]. In the context of chemoresistance, type I interferon can induce an antiviral-like response in tumor cells, potentially enhancing their resistance to chemotherapy by activating DNA damage repair mechanisms. Additionally, it modulates the immune response within the tumor microenvironment. For example, type I interferon can activate natural killer (NK) cells and cytotoxic T lymphocytes (CTLs), which can target and kill tumor cells. However, in some cases, tumor cells may develop mechanisms to evade immune-mediated killing, thereby contributing to chemoresistance [19].

Pathways such as the *Mitogen-Activated Protein Kinase* (*MAPK*) pathway interact closely with TLR-4 signaling. TLR-4 activation can trigger the phosphorylation and activation of *Extracellular Signal—Regulated Kinases* (*ERKs*), *c-Jun N-terminal Kinases* (*JNKs*), and *p38 MAPK*. For example, TLR-4 activation can induce *Ras-mediated Raf-Mitogen-Activated Protein Kinase Kinase (MEK)—ERK* phosphorylation cascades. Activated *ERKs* further phosphorylate transcription factors such as *Elk-1* and *c-Fos*, promoting the expression of genes related to cell survival, proliferation, and inflammation. Notably, *ERK* activation enhances *Cyclooxygenase-2* (*COX-2*) expression, which is crucial for prostaglandin production and contributes to the formation of a tumor-promoting microenvironment. Additionally, the *JAK-STAT3* pathway, activated by *IL-6,* also interacts with *TLR-4* signaling. *STAT3* activation upregulates anti-apoptotic proteins (e.g., *Bcl-2*) and promotes cell cycle progression through the regulation of cyclins and cyclin-dependent kinases, ultimately contributing to chemoresistance in tumor cells [6, 16–18, 20].

*TLR-4* can be activated by endogenous ligands such as heat shock proteins (HSPs), fibrinogen, and high mobility group box 1 (HMGB1) [21–23]. HSPs, produced in response to stress, act as molecular chaperones and bind TLR-4, triggering immune cell activation and cytokine production [24, 25]. Fibrinogen, essential for clotting, also interacts with *TLR-4*, activating immune responses at injury or infection sites [25]. HMGB1, released from damaged cells, binds TLR-4 with high affinity, promoting inflammation [25, 26]. These ligands stimulate signaling pathways, leading to gene transcription and antimicrobial responses [15–17]. When *TLR-4* is abnormally expressed in tumor cells, it can be activated directly by paclitaxel or indirectly by other chemotherapeutic drugs. This activation drives tumor-related inflammatory reactions, resists cell apoptosis, and ultimately enables tumor cell survival [24]. This review aims to systematically analyze *TLR-4*-mediated chemoresistance mechanisms in ovarian cancer, breast cancer, liver cancer, cervical cancer, colorectal cancer and other malignancies induced by drugs like paclitaxel, cisplatin, docetaxel (DTX), 5-fluorouracil. The findings will provide a literature basis for effectively combating tumor drug resistance.

## TLR-4 in chemoresistance in different kinds of cancer

### TLR-4 in chemoresistance in breast cancer (BC)

Extensive clinical evidence supports the overexpression of toll-like receptor family members in human BC cells. Among them, TLR-4 overexpression significantly impacts the occurrence and progression of BC. Studies show that TLR-4 was significantly up-regulated in invasive BC compared with non—malignant breast cells, and TLR4 + cells were significantly more responsive to lipopolysaccharide (LPS) stimulation than TLR4 − cells. Additionally, when TLR4 + cells were stimulated by paclitaxel (PTX), the TLR-4 expression level was positively correlated with the PTX dose, and the chemical resistance to PTX increased. Through a retrospective study of 374 patients with primary invasive BC, Saponaro et al. [25] found that TLR-4 could serve as an independent prognostic factor for evaluating the 5-year disease—free survival of BC patients. They observed that TLR-4 was closely related to the NLRP3 signaling pathway, and both could be new targets in the combined therapy of BC.

PTX, one of the main chemotherapeutic drugs for BC, induces BC cell apoptosis by over—stabilizing microtubules and preventing cell division. However, PTX can not only induce the release of inflammatory mediators and upregulate survival-promoting proteins but also act as an LPS analog to activate the TLR-4 signaling pathway, ultimately inducing BC cell chemotherapy resistance. Volk Draper et al. [26] reported that TLR-4 overexpression could reverse the PTX therapeutic effect and lead to adverse outcomes of lung and lymph metastasis. Sandeep Rajput et al. [27] reported that the growth rate of breast cancer (BC) in nude mice inoculated with breast cancer cells transfected with TLR-4 overexpression plasmids was significantly higher than that in the control group of nude mice inoculated with breast cancer cells transfected with TLR-4 silencing plasmids. After ceasing PTX treatment, nearly all nude mice inoculated with breast cancer cells transfected with TLR-4 overexpression plasmids experienced tumor recurrence in a short time. In contrast, 40% of the nude mice inoculated with breast cancer cells transfected with TLR-4 silencing plasmids remained tumor-free. The TLR-4 expression in TLR4 + cancer cells was significantly up-regulated after PTX treatment, while there was no significant change in TLR4 − cancer cells after treatment, and the TLR-4 expression level in BC cells was positively correlated with the survival rate after PTX treatment.

To enhance the therapeutic effect of PTX, researchers have explored and developed small-molecular compounds that can block the TLR-4 signal pathway [28]. Sootichote et al. [29] found that CpdA (2-(4-acetoxyphenyl)-2-chloro-N-methylethylammonium chloride) could reduce the expression levels of IL-6, IL-8, and x- linked inhibitor of apoptosis (XIAP) induced by PTX in MDA -MB-231 cells. They found that PTX significantly increased TLR-4 expression in MDA-MB-231 cells but had no significant effect on TLR4^−^ cancer cells. TLR-4 knockdown enhanced the therapeutic effect of PTX compared to control cells. Isobologram revealed a synergistic effect of CpdA and PTX. Zahra Zandi et al. [30] also reported that blocking TLR-4 with TAK-242 (also known as restorvid, which can specifically bind to the TIR domain of TLR-4) could down-regulate the expression of EGFR, c—Myc, and other genes and specifically inhibit the activity of TLR-4^+^ BC cells, ultimately achieving the goal of improving chemotherapy sensitivity [31].

These data indicate that TLR-4 is crucial for BC resistance to PTX. Thus, blocking the TLR-4 pathway can not only increase BC cell sensitivity to PTX but also enhance the PTX therapeutic effect.

### TLR-4 in chemoresistance in ovarian cancer (OC)

Malignant OC is the leading cause of death from gynecological tumors worldwide [1]. Studies show that most OC patients will develop drug-resistant clones before and after treatment. It is widely acknowledged that TLR-4 is one of the key molecules promoting OC drug resistance development.

Reports indicate that the expression of TLR-4/MyD88 is significantly associated with poor patient survival, manifested as a significant shortening of the survival period and median recurrence time [32]. The TLR-4/MyD88 signal pathway plays a critical role in the progression, metastasis, and PTX resistance of malignant OC cells. Luo et al. [33] studied the ovarian epithelial tissue of 399 patients who received standard postoperative chemotherapy with PTX and platinum, including 63 normal ovarian epithelium, 124 serous cystadenoma, 95 borderline serous adenoma, and 117 serous adenocarcinomas. They found that the TLR-4 expression level was positively correlated with tumor malignancy and chemotherapy resistance. Hun Choi et al. [34] found that an endogenous ligand of TLR-4, pancreatic adenocarcinoma up-regulated factor (*PAUF*), could induce tumor cell activation and proliferation through ERK, JNK, and p38 activation, which was positively related to chemical resistance. Charles et al. [32] transfected the chemoresistant OC cell line SKOV-3 with siRNA specifically targeting MyD88 or TLR-4 and found that silencing TLR-4 made SKOV-3 OC cells exhibit an increased-chemical-sensitivity phenotype, while the loss of MyD88 expression had no effect. Although TLR-4 is expressed in almost all ovarian epithelial cells, MyD88 seems to be expressed only in malignant ovarian tumors. A negative correlation between tumor differentiation and MyD88 expression level was observed in embryonic carcinoma (EOC), indicating that MyD88-positive cancer cells were more aggressive with higher adverse biological characteristics, such as resistance to standard platinum and taxane chemotherapy. The increased expression of miR-21 and miR-146a in MyD88 − cells suggests that they may act as negative regulators of the TLR-4/MyD88 pathway. Bates et al. [35] observed that the expression of TLR-4, MyD88, and (mitotic arrest deficient 2) MAD2 was significantly associated with poor prognosis in patients with high-grade serous ovarian cancer (HGSOC). They found that knockdown of TLR-4 expression in SKOV-3 cells could enhance cell sensitivity to PTX, while knockdown of MAD2 could significantly exacerbate PTX resistance. Inhibition of MAD2 expression in A2780, OVCAR-3, and SKOV-3 cell lines led to a significant increase in TLR-4 expression, suggesting an important potential relationship between TLR-4 and MAD2. MAD2 is associated with many cell senescence-related genes and processes, and these senescent cells may play a role in anti-PTX, protecting non-senescent cancer cell populations and providing a tumor-growth environment during chemotherapy. Huang et al. [36] used atractone (AO-1), a TLR-4 blocker, and PTX simultaneously on SKOV3 and A2780 cells and found that AO-1 could reduce the expression of IL-6, VEGF, and Survivin proteins induced by PTX and enhance the growth inhibition and early apoptosis of MyD88 + EOC cells induced by PTX, that is, enhance their chemosensitivity. It is suggested that a TLR-4 blocker combined with PTX may be an effective strategy to reduce PTX resistance and improve EOC treatment. Several experiments have confirmed that PTX and LPS had similar effects in MyD88 + EOC cells, both of which could induce the upregulation of p-AKT and XIAP, thus overcoming the pro-apoptotic effect [28, 37]. Recently, Huang et al. [38] demonstrated that the TLR-4/IL-6/IRF1 axis was crucial for PTX resistance by analyzing TLR-4 and androgen receptor (AR) regulated genes related to PTX resistance. Genistein could inhibit AR activation and reduce OC chemotherapy resistance.

In summary, TLR-4 is crucial for ovarian tumor cell chemoresistance and has the potential to be a biomarker for OC chemoresistance. However, a single TLR-4 molecule likely cannot fully reflect the pathophysiological process in OC patients. Therefore, exploring the relationship between the TLR-4 signal pathway and other markers and jointly blocking them may be highly significant for formulating a new treatment approach for OC patients.

### TLR-4 in chemoresistance in colorectal cancer (CRC)

Currently, patients with advanced CRC mainly receive adjuvant chemotherapy based on fluorouracil. Thus, overcoming 5-FU chemotherapy resistance is crucial for improving CRC prognosis. In recent years, TLR-4 overexpression in inflammatory bowel disease and colitis-associated colorectal cancer has been widely recognized. TLR-4 is closely linked to CRC invasion, metastasis, and poor prognosis and further participates in epithelial-mesenchymal transition and tumor microenvironment composition [39]. A substantial number of studies have confirmed that the TLR-4/MyD88 signal can cause excessive cell proliferation, thereby promoting colitis-associated colorectal carcinogenesis through the cyclooxygenase 2, epidermal growth factor receptor, and β-catenin-dependent pathway. Eritoran, a structural analog of the lipid A part of LPS, binds to MD2 without causing conformational changes in the TLR-4-MD2 complex. Kuo et al. [40] applied the TLR-4 antagonist eritoran in the colon of CD14 + TLR4 + wild-type mice with chemically induced CRC and found that apoptosis in tumor cells was significantly enhanced, and CRC growth was reduced. Meanwhile, an increasing number of studies have shown that the intestinal flora may affect the efficacy of anti-tumor drugs on CRC. Zhang et al. [41] found in vitro that *Fusobacterium nucleatum* (*Fn*) could up-regulate the expression of baculoviral IAP repeat-containing 3 (*BIRC3*) through the TLR-4/NF-κB pathway, ultimately reducing CRC cell chemosensitivity to 5-FU. Although the virulence factors and mechanisms of *Fn* affecting CRC development and prognosis remain incompletely clarified, it still indicates that studying the interaction mechanism between the intestinal flora and host cells can offer a new direction for reducing chemotherapy resistance.

TLR-4 can significantly reduce the efficacy of 5-FU by promoting the activation of the NLRP3 inflammasome in myeloid-derived suppressor cells (MDSCs) and macrophages within the tumor microenvironment (TME), triggering an inflammatory cascade reaction. The NLRP3 inflammasome, consisting of nucleotide-binding oligomerization domain-like receptor 3 (NLRP3), apoptosis-associated spot-like protein (ASC), and pro-caspase-1, is a protein complex with a molecular weight of approximately 700 kD that recognizes pathogen-related or damage-related molecular patterns within cells. By promoting the processing, maturation, and secretion of IL-1β and IL-18, it generates various biological effects, including drug resistance in tumor cells, by regulating the expression of inflammation-related genes. In the normal resting state, the NLRP3 inflammasome exists at a very low level. However, once its activation is uncontrolled and its expression is abnormally elevated, it will trigger an inflammatory cascade reaction, cause organ damage, and significantly reduce the efficacy of 5-FU. The activation of NLRP3 is a two-signal activation process: the first signal is the priming signal, mainly targeting the transcription and post-translational modification levels of NLRP3 components. The second signal is the activating signal, related to mitochondria, ion flow, and lysosomes, and involves the assembly of each NLRP3 inflammasome component and the initiation of related downstream signaling pathways [42]. When the TLR-4/NF-κB signaling pathway is activated by a specific ligand, bacterial-derived LPS, it mainly acts on the first signal (the priming signal). On one hand, it promotes the transcription and translation of MDSCs in the TME and NLRP3 inflammasome components in macrophages (NLRP3 and pro-IL-1β). Only in this way can the NLRP3 protein form a complex with ASC and then bind to caspase-1 to form the inflammasome [43–45]. On the other hand, TLR-4 can recruit more MDSCs and macrophages into the TME by influencing NLRP3 initiation. 5-FU acts on the second signal (the activating process) of NLRP3. After the synthesis of all NLRP3 inflammasome components, 5-FU treatment increases the permeability of the lysosome membrane in MDSCs and macrophages, and then cathepsin B is released from the lysosome through the lysosome membrane. Cathepsin B then promotes the assembly of NLRP3 inflammasome components and the synthesis and release of IL-1β. IL-1β will stimulate the secretion of IL-17 by CD4 + T cells, accelerating tumor progression and reducing the efficacy of 5-FU [46].

In conclusion, TLR-4 may be a key factor in the development of resistance to 5-FU chemotherapy in colitis-associated CRC, suggesting that the combined use of TLR-4 antagonists and regulation of the intestinal flora may provide potential ideas for further improving CRC treatment.

### TLR-4 in chemoresistance in prostate cancer (PC)

As one of the most common male malignant tumors, PC is also an important cause of cancer-related death in male. The main treatment methods for early PC include hormone therapy or androgen deprivation therapy. As the disease progresses and worsens, patients must accept DTX as the final treatment option [47]. Therefore, seeking ways to enhance the therapeutic effect of DTX and optimize its anti-tumor activity has become a research hotspot. Zhang et al. [48] confirmed that LPS binding to the TLR-4 receptor can endow PC-3 human PC cells with chemical resistance to DTX, which may be related to the activation of the phosphatidylinositol 3-kinase/protein kinase B (PI3K/Akt) pathway. Studies have found that aging PC cells can alter the tumor microenvironment by releasing high mobility group box 1 (HMGB1), one of the specific ligands of TLR-4. Zhou et al. [49] also reported that recombinant HMGB1 can rapidly activate NF-κB in DU145 tumor cells, then induce the production of sCLU (cytoplasmic lectin) and increase the tolerance of residual tumor cells to DTX. This process can be blocked by TLR-4 antagonists. In control experiments with non-tumor tissues, the expression levels of TLR-4 downstream cytokines (such as IL-6, IL-8, and IL-10) were observed to be increased in PC tissues, indicating that the response to TLR-4 ligand stimulation may be related to the persistent inflammatory response experienced by prostate cells during carcinogenesis.

In brief, reducing PC resistance to DTX chemotherapy is of great significance for metastatic castration-resistant patients, and TLR-4-targeted therapy or TLR-4 blockers may be a promising starting point.

### TLR-4 in chemoresistance in liver cancer

Hepatitis C virus, alcoholism, and obesity are widely recognized as major risk factors for hepatocellular carcinoma (HCC). Among them, obesity, alcoholism, and other pathogenic factors can increase intestinal permeability, trigger endotoxemia, and then activate liver TLR-4, inducing HCC occurrence and progression. Wang et al. [50] reported that in HCC, activated TLR-4 can promote HCC occurrence and development by regulating Ku70 expression in mice. Chen et al. [51] proved that HCC occurrence and progression in mice were TLR-4-dependent by feeding mice with liver-specific expression of HCV NS5A an alcohol or Western diet for 12 months. In subsequent clinical trials, it was found that TLR-4, NANOG, and their downstream targets Yes-associated protein 1 (YAP1) and insulin-like growth factor 2 mRNA-binding protein 3 (IGF2BP3) expression were significantly correlated with HCC patient prognosis [52]. Moreover, the relationship between TLR-4 and hepatoblastoma (HB) drug resistance has also been reported. Chih Cheng Hsiao et al. [53] found that LPS stimulation could significantly increase TLR-4 expression levels in HepG2 cells, and this effect could be strengthened by IL-8. This study improved HepG2 cell chemosensitivity to cisplatin and doxorubicin by silencing the TLR-4 gene, providing strong evidence for the regulatory role of TLR-4 in inducing HB cell chemosensitivity.

### TLR-4 in chemoresistance in other types of cancer

TLR-4 promotes melanoma cell growth and survival and is essential for inducing tumor resistance to paclitaxel (PTX). Wu et al. [54] found that cytokines such as IL-8 and vascular endothelial growth factor (VEGF) were positively correlated with melanoma progression. It was reported that treating A375 cells with Icariside II and PTX led to a greater reduction in IL-8 and VEGF than PTX alone. Sootichote et al. reported that the TLR-4 expression level in MDA-MB-435 melanoma cells treated with PTX was significantly increased. The TLR-4-mediated pathway controls IL-6 and IL-8 production and induces anti-apoptotic protein XIAP in cancer cells, leading to PTX resistance. CpdA targets this pathway, attenuating TLR-4-mediated PTX resistance in breast cancer and melanoma by inhibiting IL-6, IL-8, and XIAP, thus enhancing PTX sensitivity. The combined use of PTX and CpdA had a synergistic effect on the apoptosis of MDA-MB-435 melanoma cells induced by PTX, and the number of viable cells after combined treatment was significantly lower than that with PTX alone [29].

It is well-known that human papillomavirus (HPV) infection is a key factor in cervical cancer development. E6 and E7 proteins in high-risk HPV can cause a series of cellular changes, increase mutations, or alter TLR expression, inducing cancer progression. Mirian et al. [55] studied TLR-4 and the linker molecule *Serile Aha and TIR Motif-containing 1* (*SARM1*) in HPV-positive cervical cancer cell line HeLa cells and found that knocking out TLR-4 or SARM1 could reduce HeLa cell proliferation and viability and proved that TLR-4-knockout cells were more sensitive to cisplatin by promoting tumor cell apoptosis.

Grazia Scandura et al. used Stiniproporphyrin IX (SnPP) and TAK-242 to inhibit Heme Oxygenase-1(HO-1) and TLR-4, respectively, and found that the therapeutic effect of bortezomib (BTZ) was significantly increased. It was confirmed that activation of the TLR4/HO-1/CO axis can endow multiple myeloma (MM) cells with mitochondrial protection and resistance to BTZ. When TLR-4 is activated in MM cells, it triggers the TLR-4/HO-1/CO axis. HO-1 upregulation produces carbon monoxide (CO), which stabilizes the mitochondrial membrane potential and reduces reactive oxygen species (ROS), preventing BTZ-induced apoptosis. This axis may also modulate other pathways, such as upregulating anti-apoptotic proteins, conferring resistance to BTZ treatment [56].

Nishiguchi et al. [57] found that c-Met, HMGB1, and protocadherin beta 9 (PCDHB9) genes were differentially expressed in sensitive and drug-resistant cases of advanced gastric cancer, and their expression levels were negatively correlated with cisplatin (CDDP) sensitivity. HMGB1, a TLR-4 ligand, can induce tumor chemoresistance by activating NF-κB through the TLR4/MyD88 pathway. Upon HMGB1 binding to TLR-4, the TLR-4/MyD88 pathway activates NF-κB. Activated NF-κB translocates to the nucleus, regulating genes involved in cell survival, such as upregulating anti-apoptotic Bcl-2 and promoting drug-efflux pump expression, reducing cisplatin concentration in cells and contributing to chemoresistance [58].

Recently, the new role of TLR-4 in pancreatic cancer treatment has been reported. Zhang et al. [59] found that stimulating TLR-4 and Adenylyl Cyclase-Associated Protein 1(CAP1) receptors with resistin (a factor secreted by macrophages in the tumor microenvironment) can activate the STAT3 pathway and endow pancreatic cancer cells with resistance to drug therapy. Resistin binding to TLR-4 and CAP1 receptors activates the STAT3 pathway. Phosphorylated STAT3 dimerizes, translocates to the nucleus, and binds to target genes. It upregulates genes for cell survival (such as Bcl-xL) and proliferation (such as c-Myc) and may enhance drug-metabolism and efflux protein expression, reducing drug concentration in cells and causing chemoresistance [60].

Martin-Medina et al. [61] emphasized the synergistic effect of the TLR family and WNT signaling in lung cancer progression. Activation of these two signaling pathways leads to the massive release of pro-inflammatory mediators such as IL-6 and Monocyte Chemoattractant Protein-1(MCP-1), ultimately inducing immunosuppression and chemotherapy resistance. Activation of TLR and WNT signaling pathways occurs through ligand-receptor binding. TLR-4 activation can trigger NF-κB, promoting IL-6 and MCP-1 production. WNT signaling activates β-catenin for gene regulation. The released cytokines attract immune cells, creating an immunosuppressive environment. IL-6 can activate the STAT3 pathway in cancer cells, upregulating anti-apoptotic and drug-efflux proteins, contributing to chemotherapy resistance.

## Conclusion

**Fig. 1:**
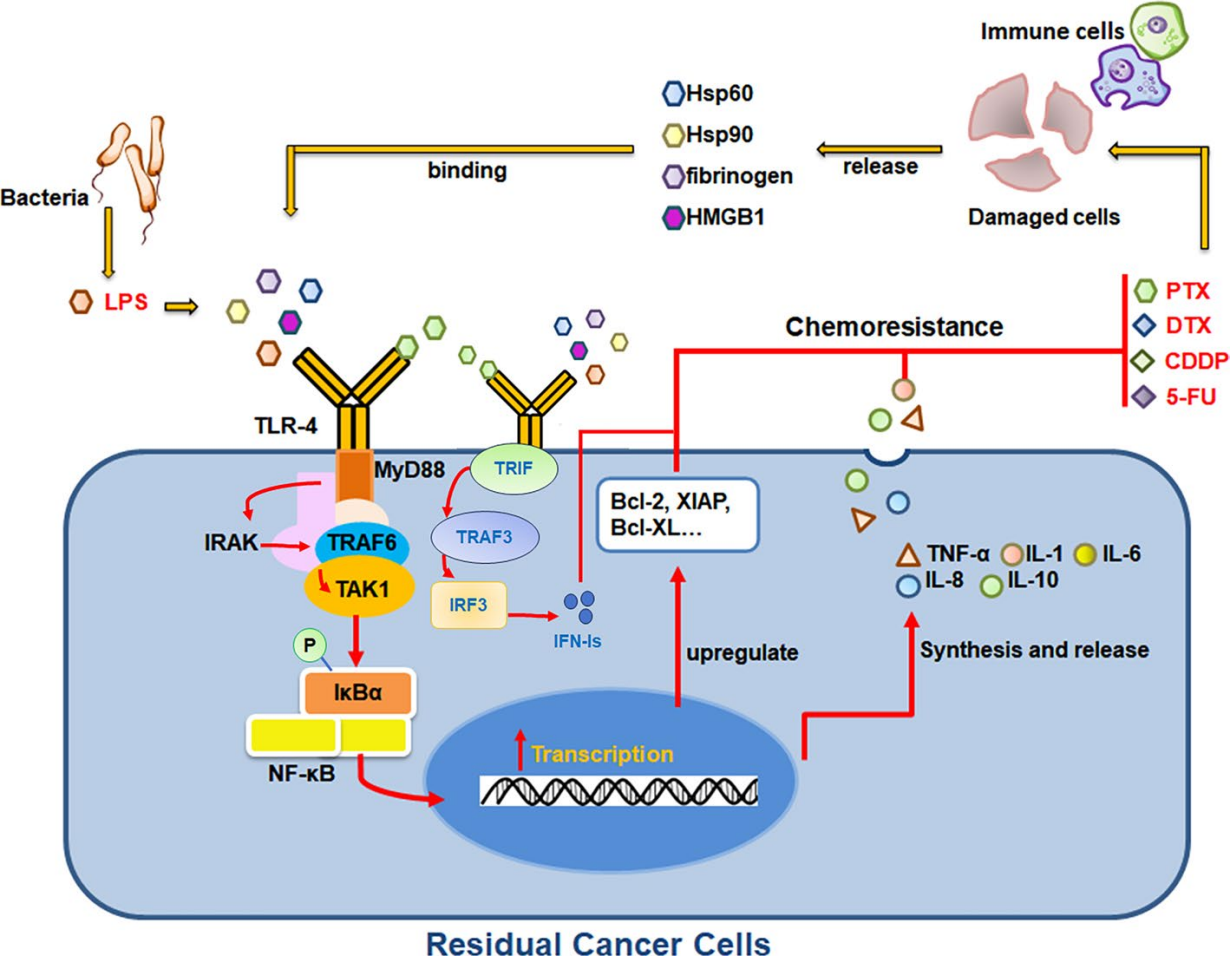
The role of chemotherapy-induced TLR-4 activation in drug resistance in cancer cells. TLR-4 recognizes pathogen-associated molecular patterns (PAMPs), such as lipopolysaccharide (LPS), and chemotherapy-induced damage-associated molecular patterns (DAMPs), including high-mobility group box 1 (HMGB1) and heat shock protein 90 (Hsp90). Paclitaxel (PTX) directly binds to TLR-4, whereas docetaxel (DTX), cisplatin (CDDP), and 5-fluorouracil (5-FU) activate TLR-4 through DAMPs released by damaged cells. TLR-4 activation triggers the following pathways: 1. MyD88-dependent pathway: MyD88 recruits interleukin-1 receptor-associated kinase (IRAK), which activates the tumor necrosis factor receptor-associated factor 6 (TRAF6)-transforming growth factor-beta-activated kinase 1 (TAK1)-IκB kinase (IKK) cascade. This leads to IκBα degradation and nuclear translocation of nuclear factor-kappa B (NF-κB). NF-κB upregulates anti-apoptotic proteins (e.g., Bcl-2) and pro-inflammatory cytokines (e.g., IL-1/IL-6); 2. MyD88-independent pathway: TRIF activates the tumor necrosis factor receptor-associated factor 3 (TRAF3)-interferon regulatory factor 3 (IRF3) axis, inducing type I interferons (e.g., IFN-α/β). Both pathways synergistically contribute to chemoresistance by enhancing tumor cell survival and promoting immune evasion

This article reviews the critical role of TLR-4 and its signaling pathways in inducing chemotherapy resistance in tumor cells (Fig. 1; Table 1). Recent clinical and laboratory studies on different types of tumors (such as breast cancer, ovarian cancer, colorectal cancer, and cervical cancer) show that commonly used anti-cancer drugs (such as paclitaxel, platinum, docetaxel, and 5-fluorouracil) have dual effects of cytotoxicity and induction of chemotherapy resistance in tumors. Tumor resistance to chemotherapy drugs can manifest as tumor cell survival, new blood vessel generation, and enhanced tumor metastasis, which are key factors affecting the therapeutic effect [62]. As a member of the toll-like receptor family, TLR-4 is involved in the first-line defense of the innate immune system formed by the body's mucous membranes, playing a role in pathogen recognition and inducing an innate immune response in the fight against inflammation and cancer [24]. Therefore, compared with other tissues, the mucous epithelium is more likely to be stimulated by inflammatory factors to activate the TLR-4 signaling pathway and is also more prone to developing inflammation-associated tumors. Summarizing the data collected so far, there is indeed more evidence of chemotherapeutic resistance in epithelial neoplasms than in sarcomas. The relevant literature is hereby summarized to help deepen the understanding of TLR-4-induced tumor resistance and provide potential new strategies for identifying tumor resistance and improving treatment regimens.

**Table 1:** TLR-4 is involved in chemoresistance in different types of cancer

Cancer type	Investigated subjects (models/cell lines)	Drug resistance	Key mechanisms of TLR-4-mediated chemoresistance
Breast cancer	MDA-MB-231 cells, nude mouse xenografts	Paclitaxel (PTX)	1. TLR-4/NF-κB → IL-6/IL-8/XIAP [17, 18] 2. NLRP3 inflammasome [19–21] 3. STAT3 synergy [25–31]
Ovarian cancer	SKOV-3, A2780 cells	Paclitaxel (PTX)	1. TLR-4/MyD88 → PI3K/AKT [22–24] 2. PAUF ligand → ERK/JNK/p38 [25, 26] 3. miR-21/146a [28, 32–38]
Colorectal cancer	MDSCs, *F. nucleatum*-infected cells	Cisplatin (CDDP)	1. Cathepsin B → NLRP3 [30] 2. *Fn*-TLR4/NF-κB/BIRC3 [31, 39–42] 3. IL-1β/IL-17 loop [43–46]
Prostate cancer	PC-3, DU145 cells	5-Fluorouracil (5-FU)	1. LPS-TLR4 → PI3K/AKT [38, 47] 2. HMGB1 → sCLU [39, 48] 3. IL-6/8/10 ↑ [47]
Liver cancer	HepG2, HCV NS5A model	Docetaxel (DTX)	1. TLR4-Ku70-YAP1 [41–43] 2. LPS/IL-8 → TLR4 [50–53]
Melanoma	A375, MDA-MB-435 cells	Cisplatin (CDDP)	1. TLR4 → IL-8/VEGF [19, 44] 2. XIAP anti-apoptosis [54]
Cervical cancer	HeLa cells	Paclitaxel (PTX)	1. HPV E6/E7-TLR4/SARM1 → NF-κB [45]

Furthermore, TLR-4 can affect tumor resistance by modulating key processes within the tumor microenvironment, including epithelial-mesenchymal transition (EMT), stemness maintenance, and stromal immunosuppression [63]. Macrophages are critical components of the tumor microenvironment. In hepatocellular carcinoma (HCC), M2-polarized tumor-associated macrophages (TAMs) promote EMT and cancer cell migration via the TLR-4/STAT3 signaling pathway. HCC cells treated with M2-conditioned medium exhibit fibroblast-like morphology and enhanced motility [64]. Overexpression of TLR-4 exacerbates tumor malignancy, whereas neutralizing antibodies targeting TLR-4 can reverse these effects. Clinically, in the relapsed HCC microenvironment, both TLR-4 and SRY-box 2 (SOX2) are upregulated. LPS-activated TLR-4 signaling enhances stemness and anti-apoptotic capabilities. In vivo studies demonstrate that the TLR-4-AKT-SOX2 axis within the tumor microenvironment promotes tumor growth [65]. Fibroblasts also interact with TLR-4 in the tumor microenvironment. In prostate cancer, cancer-associated fibroblasts (CAFs) mediate TLR-4-dependent inhibition of T-cell proliferation, contributing to immunosuppression. Cinnamaldehyde alleviates this immunosuppressive effect by activating TLR-4-related pathways, such as p-JNK/p-TAK1/p-c-Jun. However, TLR-4 inhibitors abrogate the immunomodulatory effects of cinnamaldehyde, underscoring the essential role of TLR-4 in stromal immunomodulation within the tumor microenvironment [66].

Based on the established correlation between the TLR-4 signaling pathway and tumor chemotherapy resistance, future research should delve deeper into whether abnormal TLR-4 expression can serve as an independent biomarker for assessing tumor resistance in clinical treatment. Additionally, it is essential to explore whether TLR-4 inhibitors could be utilized in cancer therapy to enhance the efficacy of commonly used drugs. In this context, a comprehensive discussion of the current status of TLR-4 modulators is warranted. Some TLR-4 modulators, particularly those tested in sepsis treatment, have failed in clinical trials. For instance, eritoran, a TLR-4 antagonist, was initially designed to inhibit TLR4-mediated overactivation of the immune response in sepsis. However, sepsis represents a highly complex condition involving dysregulation of the immune system, with TLR-4 being only one component of an extensive network of inflammatory mediators. Eritoran's failure may be attributed to its inability to fully address the intricate crosstalk among various immune pathways. When TLR-4 is overactivated in sepsis, it triggers a "cytokine storm," and simply blocking TLR-4 may not restore immune balance due to the activation of compensatory pathways [67]. Similarly, in tumor cells, the blockade of TLR-4 may lead to the activation of alternative pathways that sustain cell survival and proliferation. This phenomenon can result in resistance to TLR-4 inhibitors in cancer cells, analogous to the compensatory pathways activated during sepsis when TLR-4 is inhibited [26].

Currently, several other TLR modulators remain under clinical investigation. CpG oligodeoxynucleotides (ODNs), which modulate TLR-4-related pathways to some extent despite being TLR-9 agonists, are being explored for their potential to enhance the anti-tumor immune response. However, these agents face significant limitations. CpG ODNs can induce systemic immune activation, leading to adverse effects such as cytokine release syndrome. Moreover, optimizing dosing regimens and delivery methods to target the tumor tissue precisely remains a challenge [68]. Another group of TLR modulators, small-molecule TLR-4 agonists, have shown promise in pre-clinical models. These agonists can potentially activate anti-tumor immune responses by promoting dendritic cell maturation and cytokine production. However, their translation to clinical use requires careful consideration of dosing, off-target effects, and potential immunotoxicity. For example, some small-molecule agonists may activate TLR-4 in non-tumor cells, leading to unwanted immune activation and associated side effects [69, 70].

In conclusion, while the TLR-4 signaling pathway holds great potential as a target for overcoming tumor chemotherapy resistance, significant hurdles remain. Future research should focus on developing more specific and effective TLR-4 modulators, understanding the complex interplay between TLR-4 and other signaling pathways in the tumor microenvironment, and designing rational combination therapies that can enhance the efficacy of chemotherapy while minimizing resistance. Additionally, further investigation is needed to elucidate the role of TLR-4 in different tumor subtypes and patient populations, which may enable personalized treatment strategies based on individual TLR-4 expression profiles. This comprehensive approach may ultimately lead to improved cancer treatment outcomes and better patient survival rates.

## Data Availability

No datasets were generated or analysed during the current study.
